# (*E*)-*N*-(1,3-Benzodioxol-5-yl)-1-(4-{[1-(prop-2-en-1-yl)-1*H*-1,2,3-triazol-4-yl]meth­oxy}phen­yl)methanimine

**DOI:** 10.1107/S1600536813025749

**Published:** 2013-09-21

**Authors:** Mehmet Akkurt, Aliasghar Jarrahpour, Mehdi Mohammadi Chermahini, Pezhman Shiri, Orhan Büyükgüngör

**Affiliations:** a Department of Physics, Faculty of Sciences, Erciyes University, 38039 Kayseri, Turkey; b Department of Chemistry, College of Sciences, Shiraz University, 71454 Shiraz, Iran; c Department of Physics, Faculty of Arts and Sciences, Ondokuz Mayıs University, 55139 Samsun, Turkey

## Abstract

In the title compound, C_20_H_18_N_4_O_3_, the dihedral angles between the central benzene ring and the 1*H*-1,2,3-triazole ring and the fused benzene ring are 65.34 (19) and 3.64 (18)°, respectively. The dioxole ring adopts a shallow envelope conformation, with the methyl­ene C atom displaced by 0.156 (5) Å from the other four atoms (r.m.s. deviation = 0.007Å). In the crystal, the mol­ecules are linked by C—H⋯O and C—H⋯N hydrogen bonds, generating a three-dimensional network.

## Related literature

For background to Schiff base compounds, see: Arora *et al.* (2002 ▶); Calligaris & Randaccio (1987 ▶); Macho *et al.* (2004 ▶); Singh *et al.* (2012 ▶); Tanaka & Shiraishi (2000 ▶).
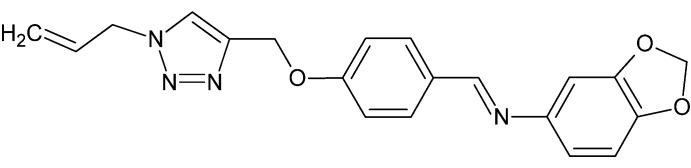


## Experimental

### 

#### Crystal data

C_20_H_18_N_4_O_3_
*M* *_r_* = 362.38
Orthorhombic, 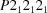

*a* = 5.1506 (6) Å
*b* = 15.334 (2) Å
*c* = 22.965 (5) Å
*V* = 1813.8 (5) Å^3^
*Z* = 4
Mo *K*α radiation
μ = 0.09 mm^−1^
*T* = 296 K
0.69 × 0.39 × 0.20 mm

#### Data collection

STOE IPDS 2 diffractometer
Absorption correction: integration (*X-RED32*; Stoe & Cie, 2002 ▶) *T*_min_ = 0.956, *T*_max_ = 0.985
13771 measured reflections
3770 independent reflections
2125 reflections with *I* > 2σ(*I*)
*R*_int_ = 0.059

#### Refinement

*R*[*F* ^2^ > 2σ(*F* ^2^)] = 0.046
*wR*(*F* ^2^) = 0.103
*S* = 0.90
3770 reflections
245 parameters
1 restraint
H-atom parameters constrained
Δρ_max_ = 0.16 e Å^−3^
Δρ_min_ = −0.12 e Å^−3^

### 

Data collection: *X-AREA* (Stoe & Cie, 2002 ▶); cell refinement: *X-AREA*; data reduction: *X-RED32* (Stoe & Cie, 2002 ▶); program(s) used to solve structure: *SHELXS97* (Sheldrick, 2008 ▶); program(s) used to refine structure: *SHELXL97* (Sheldrick, 2008 ▶); molecular graphics: *ORTEP-3 for Windows* (Farrugia, 2012 ▶); software used to prepare material for publication: *WinGX* (Farrugia, 2012 ▶) and *PLATON* (Spek, 2009 ▶).

## Supplementary Material

Crystal structure: contains datablock(s) global, I. DOI: 10.1107/S1600536813025749/hb7140sup1.cif

Structure factors: contains datablock(s) I. DOI: 10.1107/S1600536813025749/hb7140Isup2.hkl

Supplementary material file. DOI: 10.1107/S1600536813025749/hb7140Isup3.cml

Additional supplementary materials:  crystallographic information; 3D view; checkCIF report

## Figures and Tables

**Table 1:** Hydrogen-bond geometry (Å, °)

*D*—H⋯*A*	*D*—H	H⋯*A*	*D*⋯*A*	*D*—H⋯*A*
C5—H5⋯O3^i^	0.93	2.59	3.471 (4)	157
C7—H7*B*⋯N2^ii^	0.97	2.59	3.380 (5)	138

Symmetry codes: (i) 
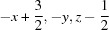
; (ii) 
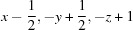
.

**Fig. 1.**
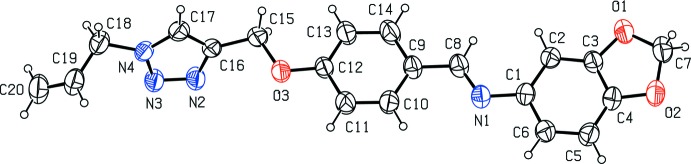
The molecular structure of compound (I) with 30% probability displacement ellipsoids.

**Fig. 2.**
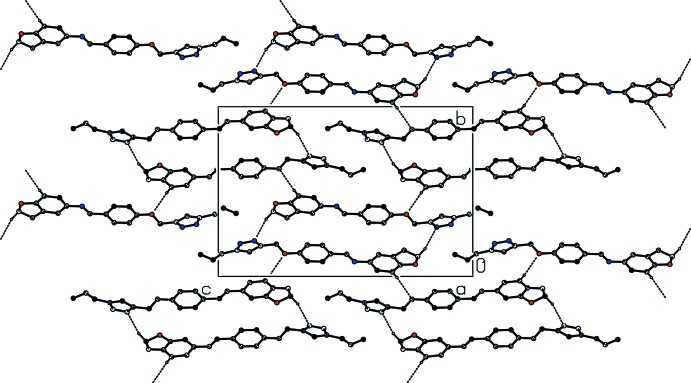
Packing diagram of compound (I) viewed down the a axis. Hydrogen bonds are indicated by broken lines. H atoms not participating in hydrogen bonding have been omitted for clarity.

**Fig. 3.**
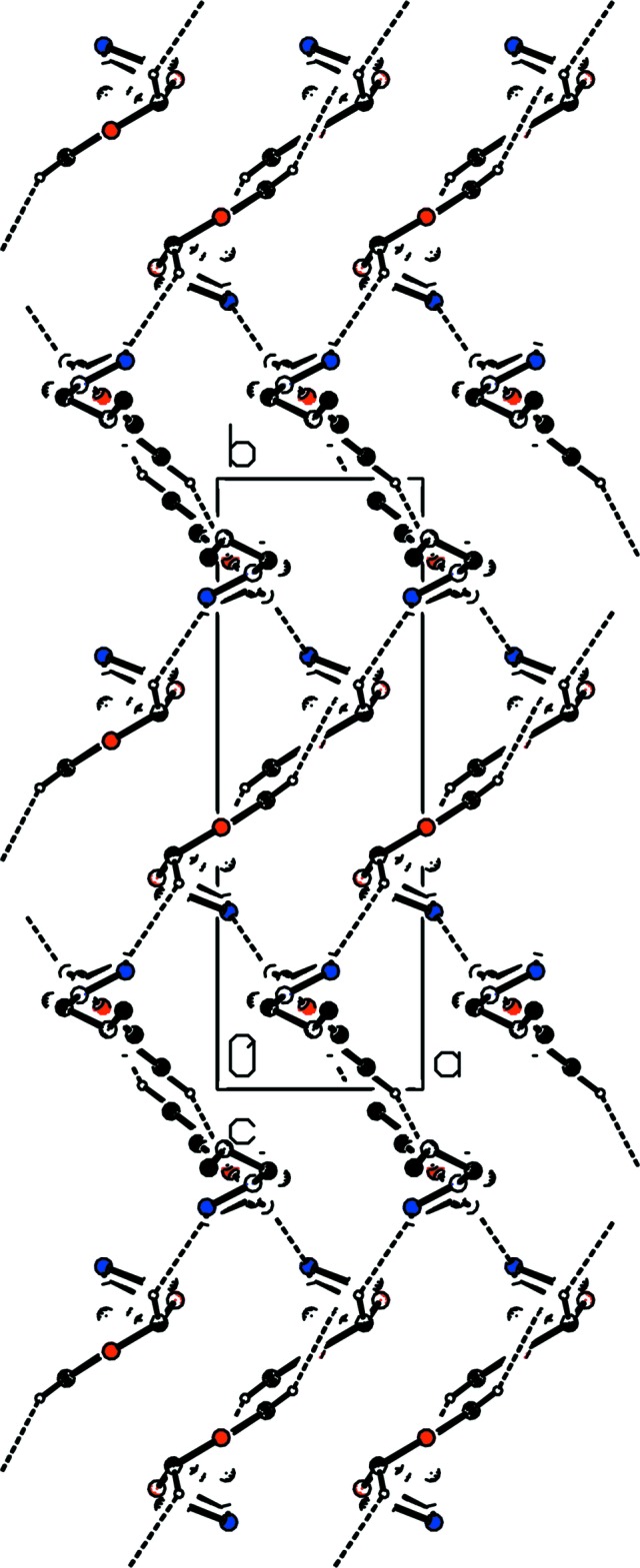
A packing diagram for compound (I), viewed down the c axis, showing the hydrogen-bonding network. H atoms not participating in hydrogen bonding have been omitted for clarity.

## supplementary crystallographic information

### 1. Comment

Schiff bases are one of the most important mixed donor systems in coordination chemistry. They result from the condensation of primary amines with carbonyl compounds to give imines containing a C=N bond (Calligaris & Randaccio, 1987). Schiff bases are widely in use for synthetic purposes both by organic and inorganic chemists (Arora *et al.*, 2002). They are used as biological, analytical, polymer and liquid crystalline materials (Tanaka & Shiraishi, 2000). Also, they are used as substrate in the preparation of a large of bioactive and industrial compounds *via* ring closure, cycloaddition, replacement reaction, cyclization and enantioselective oxidation (Macho *et al.*, 2004). Triazoles are also important class of heterocycles because of their varied biological activities (Singh *et al.*, 2012). Due to their broad range of biological activities and their value as synthetic precursors for pharmaceutical compounds, 1,2,3-triazole derivatives have received increasing attention. Therefore, compound (I), which has the two mentioned features, was synthesized and its X-ray studies is reported here.

As shown in Fig. 1, the 1*H*-1,2,3-triazole ring (N2–N4/C16/C17) and the 1,3-benzodioxole ring system (O1/O2/C1–C7) of the title compound (I) are almostly planar with maximum deviations of 0.002 (3) Å for N4 and 0.078 (5) Å for C7, respectively. The dihedral angle between the above-mentioned rings 62.91 (15)°. The benzene ring (C9–C14) in the middle of the molecule (I) forms dihedral angles of 65.34 (19) and 3.73 (16)° with the 1*H*-1,2,3-triazole ring and the 1,3-benzodioxole ring system, respectively. The C1–N1–C8–C9, C12–O3–C15–C16 and N4–C18–C19–C20 torsion angles are 179.5 (3), -176.5 (3) and 126.6 (5)°, respectively.

In the crystal, C—H···O and C—H···N hydrogen bonding interactions (Table 1; Figs. 2 & 3) connect the adjacent molecules, forming three dimensional network. However, C—H···π interactions and π-π stacking interactions are not observed.

### 2. Experimental

Reaction of 4-((1-allyl-1*H*-1,2,3-triazol-4-yl)methoxy)benzaldehyde (1.00 mmol) with benzo[*d*][1,3]dioxol-5-amine (1.00 mmol) in refluxing ethanol gave the title compound. Recrystallization from ethanol gave light brown prisms in 72% yield. Mp: 373–375 K. IR (KBr, cm^-1^):1612 (C=N). ^1^H-NMR (250 MHz, CDCl_3_) δ (p.p.m.): 4.92 (d, 2H, J=7.5 Hz), 5.21 (s, 2H), 5.30 (m, 2H), 5.91 (O—CH_2_—O, s, 2H), 5.97 (m, 1H), 6.75 (aromatic H, d, 2H, J=10 Hz), 6.99 (aromatic H, d, 2H, J=10 Hz), 7.19 (aromatic H, s, 1H), 7.81 (aromatic H, d, 2H, J=10 Hz), 7.57 (H triazole, s, 1H), 8.30 (HC═N, s, 1H). ^13^CNMR (62.9 MHz, CDCl_3_), δ (p.p.m): 62.8 (CH_2_—N), 62.1 (CH_2_—O), 101.3 (O—CH_2_—O), 101.7–148.2 (aromatic carbons and C=C triazole), 158.0 (C=N).

### 3. Refinement

All H atoms were plocated geometrically with C—H = 0.93 - 0.97 Å, and refined using a riding model with *U*_iso_(H) = 1.2*U*_eq_(C). The absolute structure was indeterminate in the present refinement. The (120), (130) and (041) reflections were omitted owing to bad agreement.

### Crystal data

**Table:**

C_20_H_18_N_4_O_3_	*F*(000) = 760
*M**_r_* = 362.38	*D*_x_ = 1.327 Mg m^−^^3^
Orthorhombic, *P*2_1_2_1_2_1_	Mo *K*α radiation, λ = 0.71073 Å
Hall symbol: P 2ac 2ab	Cell parameters from 11139 reflections
*a* = 5.1506 (6) Å	θ = 1.3–27.2°
*b* = 15.334 (2) Å	µ = 0.09 mm^−^^1^
*c* = 22.965 (5) Å	*T* = 296 K
*V* = 1813.8 (5) Å^3^	Prism, light brown
*Z* = 4	0.69 × 0.39 × 0.20 mm

### Data collection

**Table:**

STOE IPDS 2 diffractometer	3770 independent reflections
Radiation source: sealed X-ray tube, 12 x 0.4 mm long-fine focus	2125 reflections with *I* > 2σ(*I*)
Plane graphite monochromator	*R*_int_ = 0.059
Detector resolution: 6.67 pixels mm^-1^	θ_max_ = 26.5°, θ_min_ = 1.6°
ω–scans	*h* = −6→6
Absorption correction: integration (*X-RED32*; Stoe & Cie, 2002)	*k* = −19→19
*T*_min_ = 0.956, *T*_max_ = 0.985	*l* = −28→26
13771 measured reflections	

### Refinement

**Table:**

Refinement on *F*^2^	1 restraint
Least-squares matrix: full	Hydrogen site location: inferred from neighbouring sites
*R*[*F*^2^ > 2σ(*F*^2^)] = 0.046	H-atom parameters constrained
*wR*(*F*^2^) = 0.103	W = 1/[Σ^2^(*F*O^2^) + (0.0508*P*)^2^] WHERE *P* = (*F*O^2^ + 2*F*C^2^)/3
*S* = 0.90	(Δ/σ)_max_ < 0.001
3770 reflections	Δρ_max_ = 0.16 e Å^−^^3^
245 parameters	Δρ_min_ = −0.12 e Å^−^^3^

### Special details

**Table:**

Geometry. Bond distances, angles *etc*. have been calculated using the rounded fractional coordinates. All su's are estimated from the variances of the (full) variance-covariance matrix. The cell e.s.d.'s are taken into account in the estimation of distances, angles and torsion angles
Refinement. Refinement on *F*^2^ for ALL reflections except those flagged by the user for potential systematic errors. Weighted *R*-factors *wR* and all goodnesses of fit *S* are based on *F*^2^, conventional *R*-factors *R* are based on *F*, with *F* set to zero for negative *F*^2^. The observed criterion of *F*^2^ > σ(*F*^2^) is used only for calculating -*R*-factor-obs *etc*. and is not relevant to the choice of reflections for refinement. *R*-factors based on *F*^2^ are statistically about twice as large as those based on *F*, and *R*-factors based on ALL data will be even larger.

### Fractional atomic coordinates and isotropic or equivalent isotropic displacement parameters (Å^2^)

**Table:**

	*x*	*y*	*z*	*U*_iso_*/*U*_eq_	
O1	0.2051 (5)	0.1543 (2)	0.27007 (10)	0.1086 (10)	
O2	0.5181 (6)	0.07091 (18)	0.22561 (9)	0.1006 (11)	
O3	0.4365 (5)	0.13425 (16)	0.73893 (8)	0.0846 (9)	
N1	0.5539 (7)	0.08711 (18)	0.46490 (11)	0.0776 (11)	
N2	0.5549 (5)	0.2095 (2)	0.85838 (11)	0.0836 (10)	
N3	0.5512 (5)	0.19340 (19)	0.91438 (11)	0.0805 (10)	
N4	0.3268 (5)	0.15416 (17)	0.92593 (10)	0.0667 (9)	
C1	0.5363 (7)	0.08489 (19)	0.40356 (13)	0.0675 (11)	
C2	0.3460 (6)	0.1275 (2)	0.36999 (13)	0.0735 (11)	
C3	0.3620 (7)	0.1170 (2)	0.31150 (14)	0.0737 (12)	
C4	0.5479 (8)	0.0678 (2)	0.28498 (14)	0.0738 (11)	
C5	0.7368 (7)	0.0265 (2)	0.31609 (15)	0.0857 (16)	
C6	0.7253 (7)	0.0361 (2)	0.37593 (14)	0.0787 (12)	
C7	0.2850 (9)	0.1171 (3)	0.21640 (16)	0.1060 (16)	
C8	0.3987 (8)	0.1305 (2)	0.49551 (14)	0.0783 (12)	
C9	0.4109 (6)	0.1334 (2)	0.55880 (13)	0.0733 (12)	
C10	0.5886 (9)	0.0871 (3)	0.59019 (15)	0.1013 (16)	
C11	0.5948 (8)	0.0893 (3)	0.64980 (16)	0.1047 (16)	
C12	0.4170 (7)	0.1383 (2)	0.67992 (13)	0.0721 (11)	
C13	0.2412 (9)	0.1849 (3)	0.65028 (15)	0.1113 (18)	
C14	0.2367 (8)	0.1829 (3)	0.59057 (15)	0.1157 (18)	
C15	0.2705 (7)	0.1901 (2)	0.77187 (13)	0.0803 (11)	
C16	0.3326 (6)	0.1800 (2)	0.83444 (13)	0.0653 (11)	
C17	0.1889 (6)	0.1448 (2)	0.87751 (14)	0.0758 (11)	
C18	0.2532 (8)	0.1336 (3)	0.98629 (13)	0.0887 (14)	
C19	0.4671 (9)	0.0978 (3)	1.02037 (16)	0.0963 (18)	
C20	0.5422 (9)	0.1288 (3)	1.06988 (17)	0.1170 (18)	
H2	0.21630	0.16100	0.38710	0.0880*	
H5	0.86600	−0.00630	0.29810	0.1030*	
H6	0.85030	0.00850	0.39860	0.0940*	
H7A	0.15200	0.07780	0.20190	0.1270*	
H7B	0.31190	0.16260	0.18770	0.1270*	
H8	0.26940	0.16230	0.47680	0.0940*	
H10	0.70940	0.05300	0.57050	0.1220*	
H11	0.71980	0.05750	0.66990	0.1260*	
H13	0.12130	0.21880	0.67030	0.1330*	
H14	0.11300	0.21580	0.57090	0.1390*	
H15A	0.09040	0.17480	0.76490	0.0970*	
H15B	0.29610	0.25020	0.76010	0.0970*	
H17	0.02600	0.11920	0.87400	0.0910*	
H18A	0.19090	0.18630	1.00500	0.1060*	
H18B	0.11160	0.09190	0.98580	0.1060*	
H19	0.55480	0.04970	1.00550	0.1160*	
H20A	0.45840	0.17680	1.08590	0.1410*	
H20B	0.68020	0.10300	1.08950	0.1410*	

### Atomic displacement parameters (Å^2^)

**Table:**

	*U*^11^	*U*^22^	*U*^33^	*U*^12^	*U*^13^	*U*^23^
O1	0.1206 (19)	0.141 (2)	0.0642 (14)	0.042 (2)	−0.0067 (14)	0.0042 (15)
O2	0.124 (2)	0.120 (2)	0.0578 (15)	0.017 (2)	0.0055 (14)	0.0026 (12)
O3	0.1008 (16)	0.0939 (16)	0.0591 (13)	0.0306 (15)	−0.0030 (12)	−0.0015 (11)
N1	0.092 (2)	0.080 (2)	0.0608 (17)	0.0018 (17)	−0.0005 (16)	0.0038 (14)
N2	0.0768 (17)	0.112 (2)	0.0619 (16)	−0.0184 (17)	0.0002 (13)	0.0072 (15)
N3	0.0732 (17)	0.107 (2)	0.0612 (17)	−0.0200 (17)	0.0011 (14)	0.0006 (14)
N4	0.0618 (14)	0.0779 (16)	0.0605 (15)	−0.0056 (14)	0.0087 (13)	−0.0084 (14)
C1	0.080 (2)	0.0608 (19)	0.0618 (19)	−0.0081 (18)	0.0036 (17)	0.0032 (14)
C2	0.0775 (19)	0.077 (2)	0.066 (2)	0.0063 (19)	0.0018 (16)	−0.0023 (16)
C3	0.084 (2)	0.075 (2)	0.062 (2)	0.004 (2)	−0.0049 (17)	0.0055 (17)
C4	0.093 (2)	0.069 (2)	0.0595 (19)	−0.004 (2)	0.007 (2)	0.0025 (16)
C5	0.097 (3)	0.087 (3)	0.073 (2)	0.010 (2)	0.0141 (19)	0.0074 (19)
C6	0.085 (2)	0.081 (2)	0.070 (2)	0.006 (2)	0.0023 (19)	0.0122 (18)
C7	0.124 (3)	0.129 (3)	0.065 (2)	0.015 (3)	−0.010 (2)	−0.005 (2)
C8	0.098 (2)	0.073 (2)	0.064 (2)	0.005 (2)	−0.0065 (18)	0.0066 (17)
C9	0.094 (2)	0.067 (2)	0.059 (2)	0.003 (2)	−0.0048 (17)	0.0070 (16)
C10	0.120 (3)	0.120 (3)	0.064 (2)	0.050 (3)	0.007 (2)	0.005 (2)
C11	0.121 (3)	0.125 (3)	0.068 (2)	0.057 (3)	−0.001 (2)	0.007 (2)
C12	0.085 (2)	0.075 (2)	0.0562 (19)	0.014 (2)	−0.0066 (16)	0.0015 (16)
C13	0.132 (3)	0.139 (4)	0.063 (2)	0.068 (3)	−0.013 (2)	−0.011 (2)
C14	0.143 (4)	0.133 (3)	0.071 (2)	0.068 (3)	−0.021 (2)	−0.003 (2)
C15	0.078 (2)	0.095 (2)	0.0678 (19)	0.023 (2)	−0.0062 (16)	−0.0114 (17)
C16	0.0604 (18)	0.074 (2)	0.0616 (18)	0.0075 (17)	−0.0012 (15)	−0.0087 (15)
C17	0.0595 (16)	0.094 (2)	0.074 (2)	−0.0092 (17)	0.0001 (18)	−0.0173 (19)
C18	0.088 (2)	0.108 (3)	0.070 (2)	−0.008 (2)	0.0180 (19)	−0.002 (2)
C19	0.129 (4)	0.093 (3)	0.067 (2)	0.012 (3)	0.016 (3)	−0.0023 (19)
C20	0.160 (4)	0.125 (3)	0.066 (2)	0.018 (3)	0.005 (3)	−0.003 (2)

### Geometric parameters (Å, º)

**Table:**

O1—C3	1.373 (4)	C12—C13	1.339 (5)
O1—C7	1.419 (5)	C13—C14	1.372 (5)
O2—C4	1.373 (4)	C15—C16	1.480 (4)
O2—C7	1.410 (6)	C16—C17	1.348 (4)
O3—C12	1.360 (4)	C18—C19	1.459 (6)
O3—C15	1.427 (4)	C19—C20	1.292 (6)
N1—C1	1.412 (4)	C2—H2	0.9300
N1—C8	1.255 (5)	C5—H5	0.9300
N2—N3	1.310 (4)	C6—H6	0.9300
N2—C16	1.348 (4)	C7—H7A	0.9700
N3—N4	1.330 (4)	C7—H7B	0.9700
N4—C17	1.327 (4)	C8—H8	0.9300
N4—C18	1.471 (4)	C10—H10	0.9300
C1—C2	1.408 (4)	C11—H11	0.9300
C1—C6	1.382 (5)	C13—H13	0.9300
C2—C3	1.355 (4)	C14—H14	0.9300
C3—C4	1.363 (5)	C15—H15A	0.9700
C4—C5	1.363 (5)	C15—H15B	0.9700
C5—C6	1.383 (5)	C17—H17	0.9300
C8—C9	1.456 (4)	C18—H18A	0.9700
C9—C10	1.364 (5)	C18—H18B	0.9700
C9—C14	1.383 (5)	C19—H19	0.9300
C10—C11	1.370 (5)	C20—H20A	0.9300
C11—C12	1.372 (5)	C20—H20B	0.9300
			
C3—O1—C7	105.3 (3)	C18—C19—C20	124.1 (4)
C4—O2—C7	105.2 (3)	C1—C2—H2	122.00
C12—O3—C15	117.2 (3)	C3—C2—H2	122.00
C1—N1—C8	122.0 (3)	C4—C5—H5	122.00
N3—N2—C16	109.0 (3)	C6—C5—H5	122.00
N2—N3—N4	107.1 (2)	C1—C6—H6	118.00
N3—N4—C17	110.3 (2)	C5—C6—H6	119.00
N3—N4—C18	120.6 (3)	O1—C7—H7A	110.00
C17—N4—C18	128.9 (3)	O1—C7—H7B	110.00
N1—C1—C2	125.4 (3)	O2—C7—H7A	110.00
N1—C1—C6	115.2 (3)	O2—C7—H7B	110.00
C2—C1—C6	119.4 (3)	H7A—C7—H7B	108.00
C1—C2—C3	116.4 (3)	N1—C8—H8	118.00
O1—C3—C2	127.0 (3)	C9—C8—H8	118.00
O1—C3—C4	109.5 (3)	C9—C10—H10	119.00
C2—C3—C4	123.5 (3)	C11—C10—H10	119.00
O2—C4—C3	110.3 (3)	C10—C11—H11	120.00
O2—C4—C5	128.1 (3)	C12—C11—H11	120.00
C3—C4—C5	121.6 (3)	C12—C13—H13	120.00
C4—C5—C6	116.2 (3)	C14—C13—H13	120.00
C1—C6—C5	123.0 (3)	C9—C14—H14	119.00
O1—C7—O2	108.6 (3)	C13—C14—H14	119.00
N1—C8—C9	123.2 (3)	O3—C15—H15A	110.00
C8—C9—C10	122.7 (3)	O3—C15—H15B	110.00
C8—C9—C14	121.0 (3)	C16—C15—H15A	110.00
C10—C9—C14	116.2 (3)	C16—C15—H15B	110.00
C9—C10—C11	122.1 (4)	H15A—C15—H15B	108.00
C10—C11—C12	120.1 (4)	N4—C17—H17	127.00
O3—C12—C11	115.3 (3)	C16—C17—H17	127.00
O3—C12—C13	125.5 (3)	N4—C18—H18A	109.00
C11—C12—C13	119.2 (3)	N4—C18—H18B	109.00
C12—C13—C14	120.5 (4)	C19—C18—H18A	109.00
C9—C14—C13	121.9 (4)	C19—C18—H18B	109.00
O3—C15—C16	108.8 (3)	H18A—C18—H18B	108.00
N2—C16—C15	123.0 (3)	C18—C19—H19	118.00
N2—C16—C17	107.5 (3)	C20—C19—H19	118.00
C15—C16—C17	129.5 (3)	C19—C20—H20A	120.00
N4—C17—C16	106.1 (3)	C19—C20—H20B	120.00
N4—C18—C19	113.1 (3)	H20A—C20—H20B	120.00
			
C3—O1—C7—O2	−10.7 (4)	C1—C2—C3—O1	−177.6 (3)
C7—O1—C3—C2	−174.9 (4)	O1—C3—C4—C5	177.1 (3)
C7—O1—C3—C4	6.8 (4)	O1—C3—C4—O2	−0.3 (4)
C7—O2—C4—C5	176.5 (4)	C2—C3—C4—C5	−1.4 (5)
C4—O2—C7—O1	10.5 (4)	C2—C3—C4—O2	−178.7 (3)
C7—O2—C4—C3	−6.4 (4)	O2—C4—C5—C6	178.1 (3)
C12—O3—C15—C16	−176.5 (3)	C3—C4—C5—C6	1.2 (5)
C15—O3—C12—C13	−5.8 (5)	C4—C5—C6—C1	−0.5 (5)
C15—O3—C12—C11	174.6 (3)	N1—C8—C9—C14	179.4 (4)
C1—N1—C8—C9	179.5 (3)	N1—C8—C9—C10	−1.8 (6)
C8—N1—C1—C2	−1.7 (5)	C8—C9—C14—C13	178.5 (4)
C8—N1—C1—C6	178.3 (3)	C8—C9—C10—C11	−178.8 (4)
C16—N2—N3—N4	−0.3 (4)	C10—C9—C14—C13	−0.4 (6)
N3—N2—C16—C17	0.1 (4)	C14—C9—C10—C11	0.0 (6)
N3—N2—C16—C15	178.2 (3)	C9—C10—C11—C12	0.8 (7)
N2—N3—N4—C17	0.4 (3)	C10—C11—C12—C13	−1.2 (6)
N2—N3—N4—C18	−175.1 (3)	C10—C11—C12—O3	178.5 (4)
C17—N4—C18—C19	142.4 (4)	C11—C12—C13—C14	0.8 (6)
C18—N4—C17—C16	174.6 (3)	O3—C12—C13—C14	−178.8 (4)
N3—N4—C17—C16	−0.4 (4)	C12—C13—C14—C9	0.0 (7)
N3—N4—C18—C19	−43.1 (5)	O3—C15—C16—N2	70.4 (4)
C6—C1—C2—C3	0.2 (5)	O3—C15—C16—C17	−111.9 (4)
N1—C1—C6—C5	179.7 (3)	N2—C16—C17—N4	0.2 (4)
C2—C1—C6—C5	−0.3 (5)	C15—C16—C17—N4	−177.8 (3)
N1—C1—C2—C3	−179.8 (3)	N4—C18—C19—C20	126.6 (5)
C1—C2—C3—C4	0.6 (5)		

### Hydrogen-bond geometry (Å, º)

**Table:**

*D*—H···*A*	*D*—H	H···*A*	*D*···*A*	*D*—H···*A*
C5—H5···O3^i^	0.93	2.59	3.471 (4)	157
C7—H7*B*···N2^ii^	0.97	2.59	3.380 (5)	138

Symmetry codes: (i) −*x*+3/2, −*y*, *z*−1/2; (ii) *x*−1/2, −*y*+1/2, −*z*+1.
